# Editorial: Special Issue on Innovative Multi-Disciplinary Approaches for Precision Studies in Leukemia

**DOI:** 10.3389/fonc.2021.744009

**Published:** 2021-08-17

**Authors:** Sandra Marmiroli, Xu Huang, Valentina Serafin, Alison M. Michie

**Affiliations:** ^1^Cellular Signaling Unit, Department of Biomedical, Metabolic and Neural Sciences, University of Modena and Reggio Emilia, Modena, Italy; ^2^Paul O’Gorman Leukaemia Research Centre, Institute of Cancer Sciences, University of Glasgow, Glasgow, United Kingdom; ^3^Laboratory of Pediatric Oncohematology, Department of Woman’s and Child’s Health, University of Padova, Padova, Italy

**Keywords:** leukemia, bioinfomatic analysis, risk stratication, metabolism, intracellular signaling, technology, AML – acute myeloid leukemia, therapeutic targets

By investigating the role of recurrent mutations, epigenetic modifications and aberrant activation of oncogenic signaling (1–4), our understanding of leukemogenesis has advanced to the stage that precision medicine is capable of transforming diagnostic and therapeutic approaches. This Research Topic focuses on the innovative technologies that will aid in the development of a multi-dimensional integrated molecular network, enabling the prediction of therapy responses and creation of novel treatments for those in most clinical need.

Risk stratification scores in AML are important tools for clinicians and scientists alike, due to the relative ease with which biomarker panels can assist with diagnosis, prediction of prognosis, overall survival (OS) and guide appropriate treatment choices. Our work has identified a gene signature associated with the histone demethylase KDM4A, the KDM4A-9 score, which is associated with poor OS and independent of age, cytogenetic risk and mutation status (2). KDM4A-9 is highly correlative with the LSC17 score, while having no overlap, showing the collective power of integrating risk scoring systems (2, 5). In this Research Topic, risk stratification was addressed by Lu et al. through bioinformatic analysis of the publicly-available databases. The authors explored the relationship of CXCR family members in AML and found that over-expression of specific CXCR receptors correlates with FAB subtypes and AML risk stratification. Of note, CXCR2 expression was identified as an independent prognostic factor. Applying machine learning algorithms to gene expression datasets, Mosquera Orgueira et al. described a new model containing 123 variables (ST-123) that was capable of predicting survival of AML patients. Two genes were identified, namely *KDM5B* and *LAPTM4B*, that have an established role in the pathogenesis of myeloid malignancies. Similarly, Sanchez Corrales et al. reviewed machine learning potential to dissect those heterogeneities that render AML hard to tackle with effective therapies, by identifying specific leukemic populations and the biomarkers that define these populations. They propose to design precision immunotherapy strategies, accounting for the heterogeneous sub-populations characteristic of each disease.

Van Gils et al. offered a comprehensive review of the diverse, mainly non-genetic, mechanisms of resistance to chemotherapy in AML, from survival pathways mediated by the bone marrow microenvironment to altered epigenetic profiles and metabolic reprogramming. Unlike most reviews on the same subject, it focused also on AML “persisters” highlighting that they are not necessarily pre-existing but arise by distinct mechanisms and may be induced by therapy. Understanding the plasticity of these mechanisms in different patients could lead to effective personalized therapy.

Metabolic reprogramming is a key hallmark of cancer and gaining a fundamental understanding of disease biology in terms of metabolic profiling may enable personalized therapies that exploit the unique weaknesses in leukemic cells. As such, Zhang et al. analyzed publicly-available databases to investigate metabolism-related prognostic factors and identified four genes (*PLA2G4A, HMOX2, AK1, SMPD3*) that when highly expressed represented a poor prognostic predictor for AML patients. Dembitz and Gallipoli described recent evidence demonstrating the validity of developing therapies that target metabolic pathways in AML. The review highlights the differential metabolic pathways that are present in distinct AML subtypes and disease stages, which may enable truly personalized treatments for these patients, supported by FDA approval of the first metabolic inhibitors targeting IDH1/2 in AML. Further promising avenues for targeting metabolic pathways are discussed.

The impact that the microenvironmental niche has on tumor persistence is now becoming appreciated, and as such the stromal component of the leukemic niche may represent a novel therapeutic target, as has been demonstrated in B cell malignancies (6). Two reports tackled different angles of this important topic. Sletta et al. explored the role of colony stimulating factor 1R (CSF1R) within the stromal compartment. High levels of CSF1R are associated with shorter patient survival in AML and RUNX1, a key driver of AML, regulates CSF1R. This review assessed the role of CSFR1 in immunoregulation of macrophage function/polarization and discussed recent pre-clinical studies and clinical trials incorporating distinct CSFR1 inhibitors, providing evidence to support the use of CSF1R-targeted therapies in AML. Simioni et al. reviewed the role of the tumor microenvironment in acute lymphoblastic leukemia. The authors summarized the main aspects concerning experimental, pre-clinical and clinical information in the field, highlighting the potential of therapeutic strategies, which include cytokines and cytokines receptors, such as CXCL12/CXCR4, the MEK/ERK and PI3K/AKT signaling networks. The importance of oncogenic signaling is highlighted also in a review by Ratti et al., that provided an in depth description of the role of phosphoinositide (PI) lipid second messengers in the oncogenesis of AML. The authors emphasize key enzymes in PI metabolism, which are emerging as putative druggable targets in leukemia. They suggest that combining bioinformatic analysis with PI pathsway targeting can lead to new combination therapies to reprogram transcriptional output, control the cell state and attenuate uncontrolled AML cell growth.

Finally, Sun et al. present data from a clinical trial (NCT03412409) in which older patients with acute leukemia or MDS underwent haploidentical-SCT using a novel conditioning regime. Hematopoietic stem cell transplantation (SCT) represents a potential cure for acute leukemia, however as most hematological malignancies occur in older individuals, in which the myeloablative conditioning regimen prior to SCT is linked to a high risk of treatment-related mortality (TRM), this is not always possible. Incorporating reduced intensity conditioning (low dose fludarabine and reduced cyclophosphamide) in patients with acute leukemia/MDS of ≥55 years old revealed that OS, leukemia free survival, TRM, and cumulative incidence of relapse were comparable to a historical, younger cohort of patients that underwent the normal conditioning regimen. This small study suggests that a reduction in the conditioning regimen prior to SCT may be a feasible option for older acute leukemia patients.

Although the treatment of leukemia in all its forms still represents a significant challenge, these articles all highlight novel methods/strategies that can be utilized to identify targets/biomarkers that enable scientists and clinicians the opportunity to develop novel stratified diagnostics or therapeutic interventions for specific patient cohorts. As the development of ‘omics approaches is a highly competitive and fast-moving field, an ongoing challenge will be how to integrate individual approaches into a robust multi-omics-based platform that can be used as a routine clinical and diagnostic tool to form the basis of digital healthcare. Indeed, artificial intelligence-lead machine learning technologies, collecting and analyzing molecular-level ‘omics information, will represent the forefront of innovation in personalized therapy for prevention and early detection of diseases such as leukemia, coupled with precision prognosis and personalized treatment pathways.

## Author Contributions

The Editorial was drafted by SM and AM, and further edited by SM, XH, VS and AM. All authors contributed to the article and approved the submitted version.

## Funding

SM: A.MA.RI.CA. E95F21001040007, AM: Blood Cancer UK project grant Ref. 18003, VS: AIRC MFAG 2018 ID 21771.

## Conflict of Interest

The authors declare that the research was conducted in the absence of any commercial or financial relationships that could be construed as a potential conflict of interest.

## Publisher’s Note

All claims expressed in this article are solely those of the authors and do not necessarily represent those of their affiliated organizations, or those of the publisher, the editors and the reviewers. Any product that may be evaluated in this article, or claim that may be made by its manufacturer, is not guaranteed or endorsed by the publisher.
